# Genetic characterization of cysteine-rich type-b avenin-like protein coding genes in common wheat

**DOI:** 10.1038/srep30692

**Published:** 2016-08-09

**Authors:** X. Y. Chen, X. Y. Cao, Y. J. Zhang, S. Islam, J. J. Zhang, R. C. Yang, J. J. Liu, G. Y. Li, R. Appels, G. Keeble-Gagnere, W. Q. Ji, Z. H. He, W. J. Ma

**Affiliations:** 1 College of Agronomy, Northwest A & F University, Yangling 712100, Shaanxi, China; 2 Australia-China Joint Centre for Wheat Improvement, School of Veterinary & Life Sciences, Murdoch University, Perth WA 6150, Australia; 3 Crop Research Institute, Shandong Academy of Agricultural Sciences/National Engineering Laboratory for Wheat and Maize/Key Laboratory of Wheat Biology and Genetic Improvement in North Yellow and Huai River Valley, Ministry of Agriculture, 250100, Jinan China; 4 National Wheat Improvement Centre, Institute of Crop Sciences, Chinese Academy of Agricultural Sciences, 12 Zhongguancun South St, Haidian District, Beijing, China 100081

## Abstract

The wheat avenin-like proteins (ALP) are considered atypical gluten constituents and have shown positive effects on dough properties revealed using a transgenic approach. However, to date the genetic architecture of ALP genes is unclear, making it impossible to be utilized in wheat breeding. In the current study, three genes of type-b ALPs were identified and mapped to chromosomes 7AS, 4AL and 7DS. The coding gene sequence of both *TaALP-7A* and *TaALP-7D* was 855 bp long, encoding two identical homologous 284 amino acid long proteins. *TaALP-4A* was 858 bp long, encoding a 285 amino acid protein variant. Three alleles were identified for *TaALP-7A* and four for *TaALP-4A. TaALP-7A* alleles were of two types: type-1, which includes *TaALP-7A1* and*TaALP-7A2*, encodes mature proteins, while type-2, represented by*TaALP-7A3*, contains a stop codon in the coding region and thus does not encode a mature protein. Dough quality testing of 102 wheat cultivars established a highly significant association of the type-1 *TaALP-7A* allele with better wheat processing quality. This allelic effects were confirmed among a range of commercial wheat cultivars. Our research makes the ALP be the first of such genetic variation source that can be readily utilized in wheat breeding.

Bread wheat (*Triticum aestivum* L.) is the most important staple food worldwide. Its unique viscoelastic properties conferred by the storage proteins, glutenins and gliadins, account for its extensive use and multi-ethnic expressions in food preparation, reflected in a wide range of wheat-derived food products^1^,^2^. The glutenin polymers are composed of an elastic backbone, formed by high-molecular-weight (HMW) subunits, and the branches, formed by low-molecular-weight (LMW) subunits which are the main contributors to dough strength and elasticity. The monomeric gliadins, conferring dough tractability, interact with the polymeric glutenins by strong covalent and non-covalent forces^3^,^4^,^5^. The structural characteristics of proteins affect polymerization behavior through both the strategic positioning of generally conserved cysteine residues and the presence of glutamine-rich repetitive regions within the polypeptide chain^5^,^6^,^7^,^8^,^9^. Cysteines constitute only a small proportion of the amino acids of gluten proteins (about2%), yet are extremely important to the structure and functionality of gluten due to their capacity to form intra- and inter-chain disulfide bonds^10^. Non-covalent bonds (hydrogen bridges, ionic interactions, and hydrophobic bonds), characteristically formed by glutamines, are responsible for the aggregation and structural stability of gluten proteins and dough structure formation^11^,^12^.

Besides the typical gluten proteins, storage protein components also include LMW gliadins or globulins with a molecular weight below 30,000 dalton^13^,^14^,^15^. Most of these atypical gluten proteins fall into the categories of ALPs or proteins with sequences similar to the previously reported LMW gliadin monomers^14^,^15^. LMW gliadins differ from gliadins and glutenins in lacking repetitive domains, with only a short sequence of proline and glutamine residues present in the mature protein^15^. The existence of proteins related to LMW gliadins, and constituting a new family of grain prolamin proteins, has also been confirmed in barley^16^,^17^ and rye^18^. DuPont and co-workers described a protein isolated from wheat grains as ‘avenin-like’, based on partial amino sequences determined by mass spectrometry^19^. Similarly, Vensel and coworkers^20^ identified five avenin-like proteins in the proteome of albumins and globulins during early and late stages of grain development. Kan and coworkers reported two classes of cDNAs encoding two types of ALPs, namely type-a and type-b^21^. In a phylogenetic analysis of the prolamin superfamily, the ALP genes co-locate as a single cluster, with its closest neighbors being avenin of oats and the sulphur-rich proteins (α-gliadins, γ-gliadins, LMW subunits of glutenin). Furthermore, in the same study the authors observed that type-a ALPs contain 14 cysteine residues, among which eight cysteines form the characteristic conserved cysteine skeleton of the typical prolamins (similar to avenin, α-gliadins, γ-gliadins, and LMW subunits of glutenin)^22^. It is noteworthy that type-a ALPs can form seven intra-chain disulfide bonds, which is typical of monomeric LMW gliadins. Type-b ALPs contain two repetitive domains (R1, R2), each with eight cysteine residues in homologous positions to the cysteines of γ-gliadin and oats avenin protein. Type-b ALPs also exhibited some differences in cysteine distribution, with a total of 18 or 19 cysteine residues. In particular, Mamone and coworkers^23^ detected type-b ALPs in the glutenin fraction of durum wheat cultivar Svevo, while Kan and co-workers^21^ found that the two cysteines in the N-terminal domain are not conserved in various *Aegilops* species, hence suggesting that they could be involved in inter-chain linkages to polymeric subunits of glutenins. The identification of type-b ALPs was supported by the acquisition of sequences from a reasonable number of tryptic peptides matching the expected molecular weights and pI values^23^. The higher number of cysteines in type-b ALPs was expected to have a significant effect on folding and the arrangement of disulfide linkages, not only by stabilizing the molecular structure, but also by influencing glutenin polymer formation. Chen and coworkers^24^ predicted that type-b ALPs were capable of forming eight intra-molecular disulfide bonds, with three free cysteine residues involved in inter-molecular disulfide bond formation. They confirmed that type-b ALPs can notably perform as “chain branches”, increasing the probability of glutenin macro-polymer (GMP) formation and including other glutenin subunits^24^. Ma and coworkers overexpressed type-b ALPs in two transgenic wheat lines, resulting in a highly significant improve of dough mixing properties and provided strong evidence for their incorporation into gluten polymers^25^.

Until now, when selecting for dough and baking quality improvement, wheat breeders have mainly relied on the genetic variation underlying gluten proteins. The effect on dough mixing properties associated with ALPs represents a novel genetic effect that has not been utilized in a targeted way in wheat grain functionality breeding thus far. Marker-assisted selection targeting ALPs depends on both natural allelic variation of ALPs and their validated effects on dough mixing properties. The objectives of this study were to locate the type-b ALP coding genes, find the number of available alleles and quantify the allelic effects for each locus, and to develop allele-specific markers for wheat grain functionality breeding.

## Results

### Type-b ALP coding genes in *Triticum aestivum*

**Figure 1:**
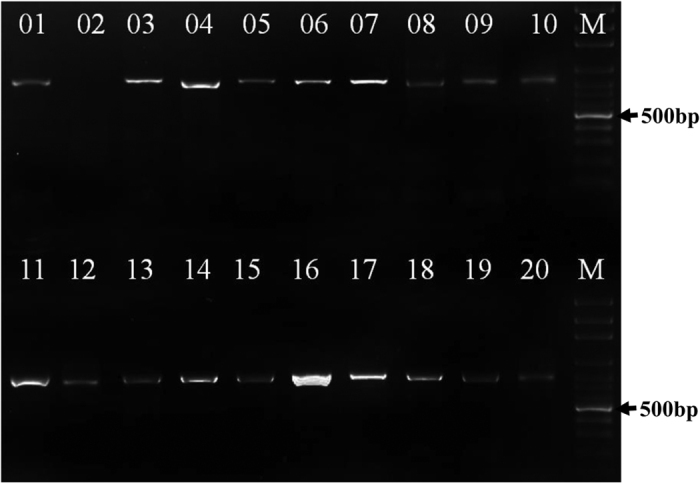
PCR amplification products of type b ALP genes from genomic DNA of 19 wheat cultivars (lines). M DNA ladder 100 bp; 01, Chara; 02, Negative control; 03, Kauz; 04, Eagle Rock; 05, Gregory; 06, Living Stone; 07, Westonia; 08, Wyalkatchem; 09, Yitpi; 10, Chinese Spring; 11, Jimai0860229; 12, Jimai13P406; 13, Jimai13J492; 14, Jimai13J492; 15, Jimai13J394; 16, Jimai13J494; 17, Jimai13P414; 18, Jimai23; 19, Jimai24; 20, Jimai44.

ALP specific primers were used to amplify the complete coding sequence of type-b ALP genes from the genomic DNA of 19 cultivars. The amplified products covering the start and stop codons were about 902 bp in length (Fig. 1). Nucleotide sequences highly similar (99%) to the previously reported type-b ALP gene sequence (Accession No. FJ529695) were obtained.

### Sequence alignment and analysis

The sequences of the amplified ALP genes were used to search the EnsemblPlants (http://plants.ensembl.org/Triticum_aestivum/Info/Index) and the International Wheat Genome Sequencing Consortium (IWGSC) databases. The results showed that type-b ALP genes were transcribed at a high rate and consisted of a single uninterrupted exon. The results were consistent with previous studies^26^. In addition, the type-b ALP gene sequences were good matches to three surveyed sequences (Chinese Spring) on chromosomes 7DS (99%), 4AL (98%) and 7AS (97%).

### Gene locations

**Figure 2:**
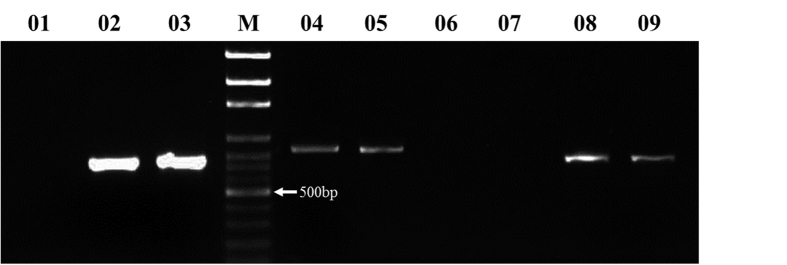
Chromosome-specific type-b ALP amplification using Chinese Spring deletion lines. M DNA ladder 100bp; 01, 7A (deletion lines); 02, 7B (deletion lines); 03, 7D (deletion lines); 04, 7A (deletion lines); 05, 7B (deletion lines); 06, 7D (deletion lines); 07, 4A (deletion lines); 08, 4B (deletion lines); 09, 4D (deletion lines).

Three pairs of specific primers were designed, targeting ALP genes on chromosomes 7AS, 4AL and 7DS to verify the blasted results (Table 1). These primers were tested across the entire set of Chinese Spring deletion lines. Results were consistent with the surveyed sequence databases and the chromosomal location of the gene products ALP-7A, ALP-4A and ALP-7D was confirmed (Fig. 2). We thus named the three ALP gene loci *TaALPb-7A*, *TaALPb-4A*, and *TaALPb-7D*, accordingly.

### SNP and indel analyses

**Figure 3:**
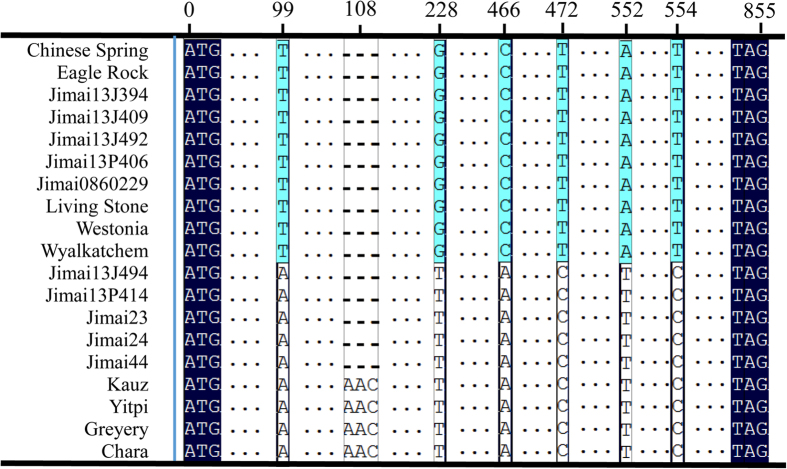
Alignment of the type-b ALP gene sequences located on wheat chromosome 7AS. SNPs/InDels are shown. Polymorphisms are represented by black box. Dashed (-) and Dots (.), respectively indicate identical and deletion nucleotides. 1, Chinese Spring; 2, Eagle Rock; 3, Jimai13J394; 4, Jimai13J409; 5, Jimai13J492; 6, Jimai13P406; 7, Jimai0860229; 8, Living Stone; 9, Westonia; 10, Wyalkatchem; 11, Jimai13J494; 12, Jimai13P414; 13, Jimai23; 14, Jimai24; 15, Jimai44; 16, Kauz; 17, Yitpi; 18, Gregory; 19, Chara.

**Figure 4:**
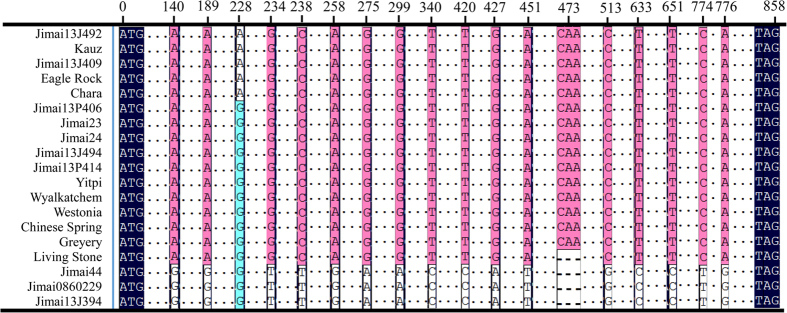
Alignment of the type-b ALP gene sequences located on wheat chromosome 4AL. SNPs/InDels are shown. Polymorphisms loci are represented by black box. Dashed (-) and Dots (.), respectively indicate identical and deletion nucleotides. 1, Jimai13J492; 2, Kauz; 3, Jimai13J409; 4, Eagle Rock; 5, Chara; 6, Jimai13P406; 7, Jimai23; 8, Jimai24; 9, Jimai13J494; 10, Jimai13P414; 11, Yitpi; 12, Wyalkatchem; 13, Westonia; 14, Chinese Spring; 15, Gregory; 16, Living Stone; 17, Jimai44; 18, Jimai0860229; 19, Jimai13J394.

**Figure 5:**
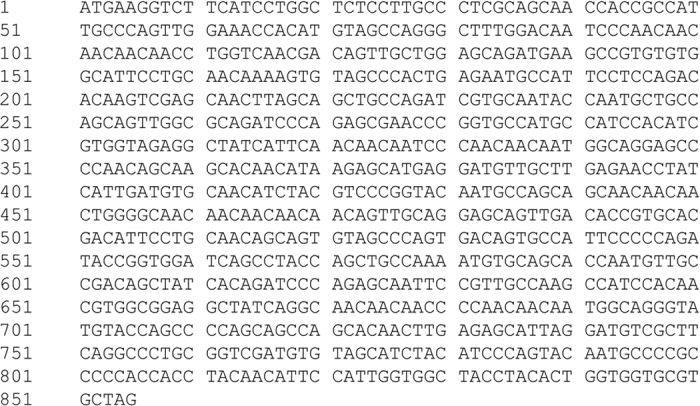
The type-b ALP gene sequences located on wheat chromosome 7DS. No SNPs/InDels are shown in 19 wheat cultivars. 1, Chinese Spring; 2, Eagle Rock; 3, Jimai13J394; 4, Jimai13J409; 5, Jimai13J492; 6, Jimai13P406; 7, Jimai0860229; 8, Living Stone; 9, Westonia; 10, Wyalkatchem; 11, Jimai13J494; 12, Jimai13P414; 13, Jimai23; 14, Jimai24; 15, Jimai44; 16, Kauz; 17, Yitpi; 18, Gregory; 19, Chara.

Genomic DNA of 19 cultivars was amplified using the primer pairs specific for ALP-7A, ALP-4A and ALP-7D, with each primer pair amplifying one single sequence across all cultivars. The full-length sequences at the three gene loci were either 855 or 858 bp, encoding proteins with 284 and 285 amino acid residues, accordingly. In addition, SNP and indel polymorphisms were discovered among the amplicons of different cultivars at loci *TaALPb*-*7A* and *TaALPb*-*4A*. Seven polymorphic sites were detected among the *TaALPb*-*7A*amplicons, including one deletion (three bases) and six SNPs involving five transversions and one transition (Fig. 3). Eighteen polymorphic sites were detected among the *TaALPb-4A* amplicons, including seventeen SNPs involving14 transversions and 3 transitions, as well as one indel variant (Fig. 4). No variationwas found at the *TaALPb-7D* locus (Fig. 5). These results indicate that multiple alleles exist for *TaALPb-7A* and *4A* while no or little genetic variation exists at the *TaALPb-7D* locus. Further comparison revealed that the *TaALPb-7A* gene had three alleles, designated *TaALPb-7A1* (GenBank accession no. KU286147), *TaALPb-7A2* (GenBank accession no. KU286148) (frequency 50.98%) and *TaALPb*-7A3 (GenBank accession no. KU286149) (frequency 49.02%) in the current study (Table 2), while *TaALPb-4A* gene had four alleles, *TaALPb-4A1* (GenBank accession no. KU286150), *TaALPb-4A2* (GenBank accession no. KU286151), *TaALPb-4A3* (GenBank accession no. KU286152), and *TaALPb-4A4* (GenBank accession no. KU286153). The *TaALPb-7D* (GenBank accession no. KU286154) locus did not show any allelic variation across the cultivars screened in this study. Analysis of the translated protein sequences revealed that the signal peptides at the N-and C-termini were rather conserved, with hardly any variation detected. The sequence differences occurred mainly in the repetitive region. Major variations were detected on 7AS and 4AL alleles. Among the 7AS alleles, *TaALPb-7A1* and*TaALPb-7A2*encode mature proteins, while allele *TaALPb-7A3* contained a stop codon (a SNP resulting in CAA→TAA codon change. Figure 3), leading to early termination of translation in 10 cultivars (Supplementary Figure 1). Anonymous silenced ALP genes have been previously reported^27^,^28^,^29^. In-frame stop codons were not detected for the 4AL alleles, although many variations occurred within the mature proteins (Supplementary Figure 2). In addition, 18 cysteine residues were detected in 7AS and 7DS ALPs. The 4AL ALPs contained 19 cysteine residues, exhibiting more cysteine residues than previously reported for endosperm-specific storage proteins.

In general, the type-b ALP proteins can be considered to be glutamine and proline-rich proteins, although less than gliadins and LMW glutenins, due to the lack of extensive repetitive sequences. At the same time, ALP proteins exhibited a conserved distribution of cysteines (Supplementary Figures 1 and 2), which are predicted to be able to form seven or eight intra-molecular disulfide bonds among the 18 or 19 cysteine residues. The remaining cysteines (at least two) may form inter-molecular disulfide bonds linking to adjacent storage protein subunits.

### Phylogenetic analysis

**Figure 6:**
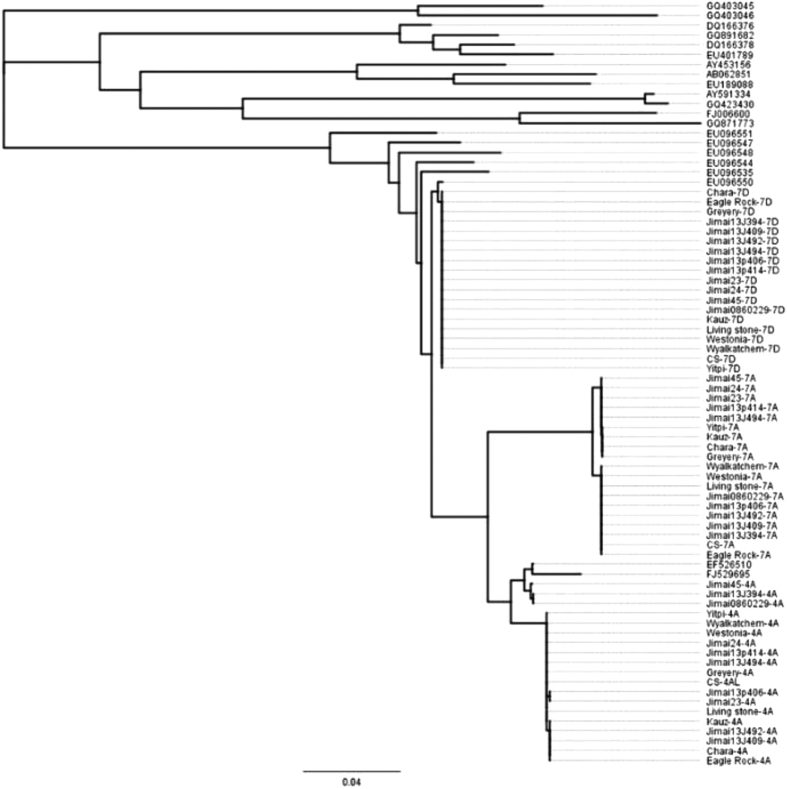
Phylogenetic analysis of the cloned sequences of type-b ALPs.

The phylogenetic relationship of the 42 cloned type b ALPs sequences was analyzed by applying UPGMA to the aligned complete coding sequences of all clones and wheat storage protein genes, as well as the reported ALPs of wheat-related species available from various databases (Table 3). As shown in Fig. 6, the cloned type-b ALP sequences clustered according to their chromosomal origin. The cloned ALP sequences were closest to the reported type-b ALP sequences of related species, followed by sequences corresponding to HMW-GS and LMW-GS, while ω-gliadin were the furthest in evolutionary terms (Fig. 6).

### Allelic effects

**Figure 7:**
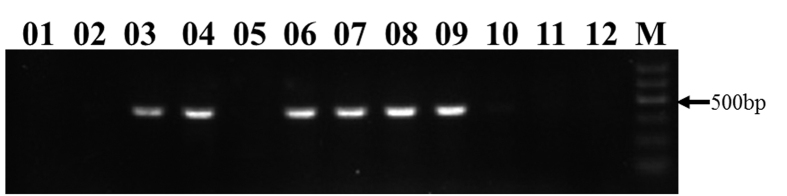
PCR amplification products of AS-PCR marker for *TaALP-7A*. M DNA ladder 100bp; 01 to 11 lanes are PCR products of selected cultivars; 12, Negative control; presence of a band indicates *TaALP-7A1* and *TaALP-7A2*, absence stands for *TaALP-7A3*.

The fact that the *TaALPb-7A* locus has two types of alleles, active and silent, allowed us to study its allelic effects. Allele-specific PCR markers were designed to differentiate the two types of *TaALPb-7A* alleles and a total 102 wheat cultivars or lines were selected for quality testing and marker analysis (Fig. 7). Mixograph analyses were conducted to assess wheat dough strength using procedures published previously^30^,^31^,^32^,^33^,^34^. Significant allelic effect differences were detected bewteen the active and silent alleles of *TaALPb-7A*. The active allele was significantly associated with higher dough strength parameters, including Midline Peak Time (P < 0.0443), and Midline Time × Width (P < 0.0096) (Table 2). Meanwhile, the component of HMW-GS, protein content and gluten content of 102 wheat cultivars or lines were analyzed (see Supplementary Table 1). Results revealed that the HMW-GS alleles were randomly distributed between the allelic types. The favorable subunit Dx5 was found in 33% of the silent wheat lines and 29% in the active lines. No significant association was detected between the allelic types and grain protein content or gluten content (Table 4), indicating the high dough strength of the active allelic type is from the expression of *TaALPb-7A*.

### Comparison of the active and silent alleles of *TaALPb*-7A at gene expression level

To further confirm the function of these two types of the *TaALPb-7A* alleles, comparison of gene expression between four Australian cultivars (Kauz, Yitpi, Gregory, and Chara) containing the active *TaALPb-7A* allele and another four wheat cultivars (Chinese Spring, Eagle Rock, Westonia, and Wyalketchem) containing the silent *TaALPb-7A* allele were conducted by using reverse transcription (RT) reaction followed by digital droplet PCR (ddPCR). Results revealed that the cultivars with the active allele give a normal gene expression, the ratios of expressed gene copy numbers between *TaALPb-7A* and actin ranged from 1:2.54 to 1:3.36 (Table 5), while the four cultivars with the silent allele had no gene expression.

## Discussion

Cysteine-rich wheat grain storage avenin-like proteins (ALPs) capable of forming intra-molecular disulfide bonds were discovered in recent years and are considered atypical gluten components of the wheat grain storage protein complement. However, the presence of similar low-molecular weight subunits in glutenins and gliadins has been reported in the 1970s^35^,^36^, and these seem capable of forming strong *in vivo* associations among themselves and with HMW-GS and LMW-GS, apparently by inter-chain disulfide bonds. ALPs make up about 1% of total endosperm proteins^37^. Contrary to the typical gluten proteins that are characterized by large repetitive central domains these non-traditional gluten proteins lack repeating sequences. In 2D gels, type-b ALPs migrate only slightly faster than the LMW glutenins, α-, or γ-gliadins, due to sequence duplication in the central domain (R1, R2), compensating to a large extent for the missing repeating sequence domain^37^,^38^. The unique properties demonstrated by type-b ALPs make them an ideal component of elastic disulfide-linked aggregates. In this study, phylogenetic analysis clearly showed that the type-b ALP sequences of common wheat clustered in the same class. The cloned sequences of the current study also clustered together, forming a small class of its own. Further, the sequences of type-b ALP genes indicated a genetic relationship to the unique C-terminal domains of gluten proteins (LMW-GS and gliadins) and are notable for the absence of significant repetitive domains of typical HMW-GS. Due to the great homology of ALP genes to gliadins and avenins, these genes might be primitive versions of earlier storage proteins predating development of the repetitive domains of the traditional gluten proteins. Alternatively, they might have evolved by losing the repetitive domains of the ancestral genes. Further work is needed to establish a clear evolutionary context for ALPs in relation to the traditional gluten proteins. It is noteworthy that almost all reported type-b ALP genes were derived from chromosome 7D, suggesting that the genes on chromosomes 7A and 4A in the current study were new discoveries. More importantly, the allelic effects identified in this study were attributed to the newly discovered 7A locus, representing a class of novel non-traditional gluten protein variation that can be readily utilized in breeding for wheat grain functionality. Our results confirmed that ALP genes belong to a multigene family, like other gluten proteins genes^26^,^39^,^40^,^41^. Cole and coworkers^42^ reported that the tetraploid forms (AABB) of wheat are actually heterogeneous for the diploid donors of the A and B genomes, which helps explain the genetic variability at the 7A and 4A loci. However, the addition of the D genome to the tetraploid ancestor of bread wheat, even though it occurred on several separate occasions, seems to have relied on the hybridization with a rather conservative *Aegilopsspp* genome^42^. We found no genetic variation at the 7D locus of type-b ALP genes in the lines and varieties investigated in this study.

Despite the potential of type-b ALP proteins to form intermolecular bonds, their low abundance and the absence of a repetitive domain might limit their ability to play a major role in determining dough functional properties, so further work is needed to establish the potential of individual ALPs for dough viscoelasticity improvement. Research conducted on transgenic type-b ALP wheat lines confirmed the presence of free cysteines capable of improving dough mixing properties by forming extra inter-chain disulfide linkages with glutenins (HMW-GS and LMW-GS)^43^. Future research on the expression of ALPs, aimed at providing a more detailed understanding of peptide chain interactions, disulfide bond arrangements, and tertiary structure formation will allow us to delve deeper into the molecular interactions with gluten proteins. Combination and association analysis using targeted allelic ALP combinations will shed further light upon the highly complex interactions due to the allelic composition of sulfur-rich proteins (γ- and α-gliadin, LMW-GS), as well as sulfur-poor proteins (ω-gliadins and HMW-GS).

Although many researchers have mentioned that type-b ALP genes of wheat belong to a multigenefamily^26^,^39^,^40^,^41^, there still remained a paucity of genetic information about the chromosomal location, number of loci and alleles, and allelic effects. The current study clearly identified the chromosomal locations of type-b ALP genes and the number of alleles at each locus for the first time. In this study, the three type-b ALP gene loci were mapped to chromosomes 7AS, 4AL and 7DS. Theoretically, due to the allohexaploid (AABBDD) nature of bread wheat, the three gene loci should be located on three homeologous chromosome locations (7AS, 7BS and 7DS). The reason for the unusual chromosomal locations can be found in the evolutionary relationships of wheat chromosome arms^44^,^45^,^46^,^47^, ie., a 4AL/7BS translocation, a pericentric inversion, and a paracentric inversion that took place in the tetraploid progenitor of hexaploid wheat^48^. This clearly provides a theoretical basis for the localization of the type-b ALP loci on 7AS, 4AL and 7DS. Common factors contribute to the different types of allelic variations, including natural evolution and artificial selection. In this study, the *TaALP-7A1* allele was detected in five Chinese cultivars (lines) (Jimai13J494, Jimai13P414, Jimai23, Jimai24 and Jimai44), while the *TaALP-7A2* allele came from four Australian cultivars (Kauz, Yitpi, Gregory and Chara). It is expected that new alleles may be discovered by expanding the number of lines and varieties screened. In addition, multilocus analyses of the experimental wheat lines have shown that striking, non-random associations of alleles develop over certain loci, *i.e.* the wheat lines develop a highly organized genetic structure featuring multilocus gene complexes. The frequency of functional alleles (50.98%) and the silent allele (49.02%) for *TaALP-7A* among eight type-b ALP alleles at three-locus combinations are found at equal levels in the tested wheat cultivars. This equal distribution of functional and non-functional alleles indicates that they could be used for marker-assisted screening for improved wheat flour processing quality. The occurrence frequency of the active *TaALP-7A1* and *TaALP-7A2* (50.98%) alleles underlines the potential utility of these alleles in wheat breeding programs.

The use of functional markers (FM) is especially important for the accurate discrimination of different alleles in marker-assisted selection (MAS)^49^,^50^. Thus far, 56 FM for processing quality traits are have been developed for 16 loci, with 62 alleles associated with HMW glutenins, LMW glutenins, polyphenol oxidase activity, lipoxygenase activity, yellow pigment content, kernel hardness, and starch properties^50^. These FMs play an important role in MAS-based breeding for improved wheat grain functionality. However, selection for wheat dough properties and breadmaking qualities has been limited to the genetic variability o gluten using the available HMW and LMW glutenin markers. The ALP allelic variation associated with dough quality discovered in the current study represents a class of novel natural genetic variation that has not been previously utilized in wheat breeding. The FM developed for the active 7AS allele can be efficiently applied to track this newly discovered variation. Important genetic and cytogenetic aspects of wheat grain functionality that still require our attention are how the expression of genes associated with dough processing properties (HMW-GS, LMW-GS, gliadins and ALPs) relates to the response of wheat storage protein accumulation to certain environmental and physiological processes.

## Methods

### Plant material and experimental design

All wheat lines used in the current study is listed in Table 6. Nineteen wheat cultivars from Australia and China were used to clone the type-b ALP genes. Field trial of 102 bread wheat cultivars (lines) with a randomized complete block design with 3 replications at the experiment station in Crop Research Institute, Shandong Academy of Agricultural Sciences, Jinan, China, in 2012 and 2013 (36°42′N, 117°4′E; altitude 48 m). Shandong has a humid subtropical climate with a mean annual rainfall of 700 mm and average maximum temperatures of over 34 °C during wheat growing season. Seeds were sown by 300 kernels per square meter in 4 × 6 m plots.

Fertilizers of 120 kg N ha^−1^, 60 kg P_2_O_5_ ha^−1^, and 120 kg K_2_O ha^−1^ were applied to the soil prior to sowing, and another 120 kg N ha^−1^ was top dressed at jointing in accordance with local wheat farming practices. The soil contained 1.64 g kg^−1^ organic matter in both years. All other standard agronomical practices were adopted. Seeds were harvested and used for mixograph, HMW-GS and NIR analysis. The means values of two trials were taken for analysis.

### DNA extraction and PCR amplification

Genomic DNA of 19 cultivars was extracted from 1-week-old seedlings by using the cetyltrimethyl ammonium bromide (CTAB) method^51^. Primers to amplify the full-length gene were designed based on the type-b ALP coding sequence from NCBI database (Accession No.FJ529695) (Table 2). The PCR conditions were set to 95 °C for 5 min, 35 cycles of 95 °C for 30 s, 60 °C for 45 s and 72 °C for 50 s, and a final extension at 72 °C for 10 min. PCR products were separated by 1.5% (w/v) agarose gel electrophoresis, and the expected fragments were purified from the gel using a Gel Extraction Kit (Promega, Madison, WI, USA). Subsequently, the purified PCR products were amplified using BigDye@version 3.1 terminator mix (Applied Biosystems) and submitted for Sanger sequencing at the Western Australia State Agricultural Biotechnology Centre. PCR and DNA sequencing were repeated three times to ensure the accuracy.

### Chromosomal locations of type-b ALP genes

The EnsemblPlants (http://plants.ensembl.org/Triticum_aestivum/Info/Index) and the International Wheat Genome Sequencing Consortium (IWGSC) databases were used to analyze the obtained sequences. After blasting the obtained sequence from 19 cultivars against the databases, good matches were found on chromosomes 7DS, 4AL and 7AS. Based on this, three pairs of specific primers were designed for each chromosome using Primer V5.0 software (http://www.premierbiosoft.com) (Table 2). Chinese Spring deletion lines were then used to test the designed chromosome-specific primers and to verify the chromosomal locations.

### Sequence analysis

The chromosome-specific primers were used to amplify the genomic DNA of 19 cultivars. The PCR products were ligated into pGEM-T Easy vector (Promega, Madison, WI, USA) following the manufacturer’s protocol and then the hybrid vector was transformed into competent cells of *E. coli* strain DH-5α. Plasmids were extracted using the Magic Mini Plasmid Prep kit (Promega, Madison, WI, USA) and the extracted DNA was amplified using BigDye^@^version 3.1 terminator mix (Applied Biosystems) for Sanger sequencing. The program Bioedit 7.0 was used for sequence analysis. Geneious^®^ software (R7) was used for multiple alignment of the translated amino acid sequences and phylogenetic analysis.

### Allele-specific marker development

Primers targeting the type-1 *TaALP-7A* allele were designed based on SNP/InDel information: F: 5′-TGCAGCAGCTTAGCAGCTGCCAT-3′; R: 5′-GCTGGT AGGCTGATCCACCGGA-3′. A total of 102 wheat cultivars and lines (Table 1) were screened using the allele-specific primers.

### HMW-GS electrophoretic analysis

The HMW-GS protein for SDS-PAGE was extracted from wheat grains by using a modified method based on Singh *et al.*^52^. In detail, 500 μl of 55% (v/v) isopropanol was mixed with crushed individual kernels for 5 min through continuous vortexing, followed by incubation (30 min at 65 C), vortexing (5 min), and centrifugation (5 min at 10000 rpm). This step was repeated three times to completely remove gliadins. Add 600 μl of 62.5 mM Tris-HCl (pH 6.8) buffer containing 10% (w/v) glycerol, 2% (w/v) sodium dodecyl sulfate (SDS), 0.003% (w/v) bromophenol blue, and 5% β-mercaptoethanol. The samples were boiled for 2 hours and then centrifuged for 5 minutes at 10000 rpm, 15 ml of upper solution of each sample were loaded on to the gel. Proteins were separated by SDS-PAGE according to Jackson *et al.*^53^ using stacking separation gels containing 4% acrylamide, 0.3% bis acrylamide, 0.1% SDS, and 0.125 M Tris-HCL (pH 6.8), and 8.7% acrylamide, 0.3% bis acrylamide, 0.1% SDS, and 0.38 M Tris-HCL (pH 8.8). The bands of HMW-GS on SDS-PAGE were scored according to the nomenclature system described by Payne and Lawrence^54^.

### Quality testing

A 10-gram mixograph (National Manufacturing Co., Lincoln NE) was used to evaluate wheat dough mixing properties, as described by Zhang and coworkers^55^. Mixograph Peak Time (MPT, min), Peak Integral (MPI, cm^2^), Peak Width (MPW, %), and Midline Time × Width (MT × W, min) were measured as the four parameters selected for evaluating the dough quality. The statistical significance of mixograph data was assessed performing T-tests using the SAS/STAT System software, Version 8.0 (SAS Institute Inc. Cary, NG)^55^. DA7200 near infrared apparatus (Perten, Swedish) was applied to analyze the protein content and gluten content following the manufacture’s suggestion.

### RNA extraction and ddPCR

RNA was extracted from 2 mature wheat grains of the 8 cultivars: Kauz, Yitpi, Gregory, Chara, Chinese spring, Eagle rock, Westonia, and Wyalketchem using the Qiagen RNeasy mini kit. The *TaALP-7A* RNA was carried out using the SuperScript^®^ II Reverse Transcriptase (Applied Biosystems), with the 3′ primer: 5′-GCTGGTAGGCTGATCCACCGGA-3′ for the active allele and 3′ primer 5′-GCTGGT AGGCTGATCCACCAGT-3′ for the silent allele. The ddPCR was performed in a QX200 ddPCR system (Bio-Rad). The forward primers were 5′-TGCAGCAGCTTAGCAGCTGCCAT-3′ for the active allele and 5′-TGCAGCAGCTTAGCAGCTGCCAG-3′ for the silent allele. The beta actin primers (F: 5′-AGAGCTACGAGCTGCCTGAC-3′; R: 5′-AGCACTGTGTTGGCGTACAG-3′) were used as the reference gene in a separate ddPCR. The ratios of the expressed gene copies between actin and TaALP-7A were calculated.

## Additional Information

**How to cite this article**: Chen, X. Y. *et al.* Genetic characterization of cysteine-rich type-b avenin-like protein coding genes in common wheat. *Sci. Rep.* **6**, 30692; doi: 10.1038/srep30692 (2016).

## Supplementary Material

Supplementary Information

## Figures and Tables

**Table 1:** Chromosome-specific primer sets for cloning type-b ALP genes.

Marker	Primer sequences (5′-3′)	Tm (°C)	Product size (bp)	Amplified target
ALP	F: TGCCACACATGATGATGCATG	60	912	Full length
	R:ATGAAGGTCTTCATCCTG GCTC			
ALP-7A	F: ATGCCAACATCAACAACCG	55	762	7A genome-specific
	R: TAGTACGCACCACCAGGGTAA			
ALP-4A	F: TCGGACAATACCAACAACAG	55	777	4A genome-specific
	R: TCTAGCATGCACCACTAGTGC			
ALP-7D	F: ATGAAGGTCTTCATCCTGGCT	58	805	7D genome-specific
	R: CTAGCACGCACCACCAGT			

**Table 2:** Mixograph parameters investigated for active and silent alleles of *TaALPb-7A.*

	Mean ± SD	
Mixograph Parameters	Band type: 0 (Frequency: 49.02%)	Band type: 1 (Frequency: 50.98%)
Midline Peak time (min)	2.32 ± 0.85	2.84 ± 1.63*
Midline Peak integral (cm^2^)	89.06 ± 35.15	107.49 ± 63.47
Midline Peak width (%)	17.36 ± 3.05	17.16 ± 3.56
Midline Time × width (%)	5.43 ± 2.22	7.03 ± 3.70***

Band type 0: silent alleles; Band type 1: active alleles.

*Difference significant at 5% probability level; ***Difference significant at 0.1% probability level.

**Table 3:** The reported nucleotide sequences of the wheat storage protein sequences and ALPs sequences of related species for phylogenetic analysis.

Gene name	Category	GenBank accession No.	Source
HMW-GS	x-type	GQ403045	*Ae. markgrafii*
	y-type	GQ403046	*Ae. markgrafii*
LMW-GS	i-type	AY453156	*T. aestivum*
	s-type	EU189088	*T. aestivum*
	m-type	AB062851	*T. aestivum*
Gliadin	α-type	EU401789	*T. turgidum*
		GQ891682	*T. aestivum*
	β-type	DQ166376	*T. aestivum*
		DQ166378	*T. aestivum*
	γ-type	FJ006600	*T. aestivum*
		GQ871773	*T. aestivum*
	ω-type	AY591334	*T. aestivum*
		GQ423430	*T. aestivum*
ALPs	Type b	EU096535	*Ae. triuncialis*
		EU096544	*Ae. juvenalis*
		EU096550	*H. vulgare*
		EU096551	*O. sativa*
		EF526510	*T. aestivum*
		FJ529695	*T. aestivum*
		EU096547	*T. monococcum*
		EU096548	*T. turgidum*

**Table 4:** Statistical analysis of Mixograph and NIR parameters.

Allele	Number of accessions	Mean Protein	Mean MTxW	Mean MPW	Mean MPI	Mean MPT	Mean Gluten
Active	52	11.68a	7.03a	17.16a	107.49a	2.84a	39.81a
Silent	50	11.98a	5.43b	17.36a	89.06a	2.32b	40.06a

(a and b indicate significance at P = 0.05).

**Table 5:** Comparison of the active and silent alleles of *TaALP-7A* at gene expression by ddPCR.

Cultivar	*TaALP-7A* type	Expressed gene copies	Mean	Actin expressiod copies	Mean	Actin/TaALP
Chinese spring 1	Silent	—	0	189056	172763	0
Chinese spring 2		—		161358		
Chinese spring 3		—		167877		
Eagle rock1	Silent	—	0	93603	92536	0
Eagle rock2		—		93141		
Eagle rock3		—		90864		
Westonia1	Silent	—	0	99825	91934	0
Westonia2		—		75655		
Westonia3		—		100321		
Wyalketchem1	Silent	—	0	20577	21489	0
Wyalketchem2		—		21837		
Wyalketchem3		—		22054		
Chara1	Active	12419	12648	38018	38794	3.07
Chara2		12921		43240		
Chara3		12605		35123		
Greygory1	Active	4872	4939	11761	12542	2.54
Greygory2		5766		15829		
Greygory3		4179		10038		
Kauz1	Active	4305	4111	6558	10865.4	2.64
Kauz2		4263		10443		
Kauz3		3767		15595		
Yitip1	Active	152806	147921	537329	497029	3.36
Yitip2		124735		521603		
Yitip3		166223		432155		
NTC1	—	—	—	—	—	—
NTC2	—	—	—	—	—	—
NTC3	—	—	—	—	—	—

**Table 6:** Name and origin of 111 wheat cultivars and advanced lines.

Name	Origin	Name	Origin	Name	Origin	Name	Origin
JimaiH101	CHINA	JimaiT112	CHINA	JimaiC70218	CHINA	Jimai13J390	CHINA
JimaiH102	CHINA	JimaiT118	CHINA	JimaiC70223	CHINA	Jimai13P406	CHINA
JimaiH105	CHINA	JimaiT120	CHINA	JimaiC70228	CHINA	Jimai13J407	CHINA
JimaiH106	CHINA	JimaiD101	CHINA	JimaiC70231	CHINA	Jimai13J408	CHINA
JimaiH107	CHINA	JimaiD102	CHINA	JimaiC70241	CHINA	Jimai13J492	CHINA
JimaiH108	CHINA	JimaiD103	CHINA	JimaiC70245	CHINA	Jimai13J424	CHINA
JimaiH109	CHINA	JimaiD104	CHINA	JimaiC70247	CHINA	Jimai13J427	CHINA
JimaiH110	CHINA	JimaiD105	CHINA	JimaiC70285	CHINA	Jimai13J394	CHINA
JimaiH111	CHINA	JimaiD106	CHINA	JimaiC70298	CHINA	Jimai13J464	CHINA
JimaiH112	CHINA	JimaiD107	CHINA	JimaiC70321	CHINA	Jimai13J467	CHINA
JimaiH113	CHINA	JimaiD108	CHINA	JimaiC70356	CHINA	Jimai13J490	CHINA
JimaiH114	CHINA	JimaiD109	CHINA	JimaiC70361	CHINA	Jimai13J492	CHINA
JimaiH117	CHINA	JimaiD111	CHINA	JimaiC70365	CHINA	Jimai13J494	CHINA
JimaiH118	CHINA	JimaiD113	CHINA	JimaiC70373	CHINA	Jimai13J495	CHINA
JimaiH120	CHINA	JimaiD116	CHINA	JimaiC70421	CHINA	Jimai9088	CHINA
JimaiH122	CHINA	JimaiD117	CHINA	JimaiC70445	CHINA	Jimai23	CHINA
JimaiH123	CHINA	JimaiD118	CHINA	JimaiC70459	CHINA	Jimai24	CHINA
JimaiH124	CHINA	JimaiD119	CHINA	JimaiC70483	CHINA	Jimai0860229	CHINA
JimaiH125	CHINA	JimaiD120	CHINA	JimaiC70509	CHINA	Chinese Spring	CHINA
JimaiT102	CHINA	JimaiD121	CHINA	JimaiT30005	CHINA	Kauz	AUSTRALIA
JimaiT103	CHINA	JimaiD122	CHINA	JimaiT40097	CHINA	Eagle Rock	AUSTRALIA
JimaiT104	CHINA	JimaiD123	CHINA	JimaiT40098	CHINA	Chara	AUSTRALIA
JimaiT105	CHINA	JimaiD124	CHINA	JimaiT40103	CHINA	Wyalkatchem	AUSTRALIA
JimaiT216	CHINA	Jimai13P307	CHINA	JimaiT40271	CHINA	Gregory	AUSTRALIA
JimaiT108	CHINA	Jimai13P406	CHINA	JimaiT40284	CHINA	Living Stone	AUSTRALIA
JimaiT109	CHINA	Jimai44	CHINA	JimaiT40362	CHINA	Yitpi	AUSTRALIA
JimaiT110	CHINA	Jimai13P414	CHINA	JimaiT40368	CHINA	Westonia	AUSTRALIA
JimaiT111	CHINA	JimaiC70107	CHINA	Jimai13J386	CHINA	—	—
