# Supplementary material for: Genetic characterization of cysteine-rich type-b avenin-like protein coding genes in common wheat
Source: Sci Rep. 2016 Aug 9;6:30692. doi: 10.1038/srep30692 (PMC4977551; doi:10.1038/srep30692)
Supplement: Supplementary Information [file srep30692-s1.doc]

**Genetic characterization of cysteine-rich type-b avenin-like protein coding genes in common wheat**

**X. Y. Chen ^1,2,3^*, X. Y. Cao^3^*, Y. J. Zhang^2^, S Islam^2^, J. J. Zhang^2^, R. C. Yang^2^, J. J. Liu^3^, G. Y. Li^3^, R Appels^2^, G. Keeble-Gagnere^2^, W.Q. Ji^1^†, Z. H. He^4^† & W. J. Ma^2^†**

^1^College of Agronomy, Northwest A & F University, Yangling 712100, Shaanxi, China.

^2^Australia-China Joint Centre for Wheat Improvement, School of Veterinary & Life Sciences, Murdoch University, Perth WA 6150, Australia.

^3^Crop Research Institute, Shandong Academy of Agricultural Sciences/National Engineering Laboratory for Wheat and Maize/Key Laboratory of Wheat Biology and Genetic Improvement in North Yellow and Huai River Valley, Ministry of Agriculture, 250100, Jinan China,

^4^.National Wheat Improvement Centre, Institute of Crop Sciences, Chinese Academy of Agricultural Sciences, 12 Zhongguancun South St, Haidian District, Beijing, China 100081

* These authors contributed equally to this work

**†**Author for communication: email: w.ma@murdoch.edu.au, zhhecaas@163.com & jiwanquan2003@126.com.

**Supplementary table. Quality parameters and HMW-GS between active and silent alleles of *TaALPb-7A***.

| **Name** | **Allele**  **(silent vs active)** | **Midline Peak time (min)** | **Midline Peak integral (cm^2^)** | **Midline Peak width (%)** | **Midline Time x width (%)** | **Protein (%)** | **Gluten (%)** | **HMW-GS** | | |
| --- | --- | --- | --- | --- | --- | --- | --- | --- | --- | --- |
|  |  |  |  |  |  |  |  | ***Glu-A1*** | ***Glu-B1*** | ***Glu-D1*** |
| Jimai13J386 | 0 | 3.39 | 120.073 | 15.349 | 8.142 | 11.35 | 35.1 | 1 | 7+8 | 5+10 |
| Jimai13J390 | 0 | 2.07 | 73.087 | 15.858 | 5.964 | 11.26 | 33.9 | 1 | 7+8 | 4+12 |
| Jimai13J394 | 0 | 1.91 | 75.477 | 17.8 | 3.386 | 12.05 | 37.9 | 1 | 7+8 | 5+10 |
| Jimai13J406 | 0 | 2.98 | 112.393 | 18.544 | 10.417 | 11.92 | 38 | 1 | 7+9 | 5+10 |
| Jimai13J407 | 1 | 4.36 | 144.533 | 12.451 | 9.479 | 11.83 | 37.4 | 1 | 7+9 | 5+10 |
| Jimai13J408 | 1 | 4.22 | 145.861 | 13.549 | 8.861 | 12.28 | 38.6 | 1 | 7+8 | 5+10 |
| Jimai13J409 | 0 | 2.25 | 79.475 | 13.999 | 4.13 | 11.73 | 36.5 | 1 | 7+8 | 5+10 |
| Jimai13J424 | 1 | 2.01 | 70.853 | 15.307 | 4.039 | 11.55 | 36.3 | 1 | 7+8 | 5+10 |
| Jimai13J427 | 0 | 1.92 | 75.136 | 16.206 | 5.948 | 11.73 | 36.9 | 1 | 7+8 | 4+12 |
| Jimai13J464 | 0 | 1.53 | 58.297 | 15.013 | 5.201 | 11.86 | 37.8 | 1 | 7+8 | 4+12 |
| Jimai13J467 | 0 | 1.63 | 60.166 | 15.632 | 5.018 | 12.51 | 40.3 | 1 | 7+8 | 4+12 |
| Jimai13J490 | 1 | 1.65 | 56.277 | 13.668 | 3.619 | 11.22 | 33.3 | 1 | 7+8 | 2+12 |
| Jimai13J492 | 0 | 1.44 | 51.338 | 15.009 | 2.777 | 10.37 | 29.4 | 1 | 7+8 | 2+12 |
| Jimai13J494 | 1 | 1.65 | 56.277 | 13.668 | 3.619 | 12.05 | 37.9 | 1 | 7+8 | 4+12 |
| Jimai13J495 | 0 | 1.88 | 61.142 | 14.373 | 4.677 | 10.12 | 28.5 | null | 7+8 | 4+12 |
| Jimai13P307 | 0 | 1.31 | 50.239 | 16.542 | 3.643 | 12.55 | 40.3 | 1 | 7+8 | 2+12 |
| Jimai13P406 | 0 | 1.66 | 61.939 | 13.43 | 4.234 | 10.96 | 35.7 | null | 7+8 | 4+12 |
| Jimai13P414 | 1 | 1.44 | 52.668 | 15.169 | 4.373 | 11.54 | 38.2 | null | 7+8 | 4+12 |
| Jimai229 | 0 | 4.94 | 206.879 | 17.379 | 11.465 | 11.8 | 39.1 | 1 | 7+8 | 5+10 |
| Jimai23 | 1 | 4.91 | 194.427 | 17.573 | 9.736 | 11.88 | 41.6 | null | 7+8 | 5+10 |
| Jimai24 | 1 | 5.32 | 181.99 | 14.329 | 10.722 | 11.93 | 42 | 1 | 7+8 | 5+10 |
| Jimai44 | 1 | 10.61 | 416.879 | 17.873 | 19.779 | 13.52 | 47.5 | 1 | 7+8 | 5+10 |
| Jimai9088 | 1 | 5.32 | 193.658 | 16.984 | 9.955 | 11.8 | 39.1 | 1 | 7+8 | 5+10 |
| JimaiC70107 | 0 | 1.67 | 58.305 | 15.633 | 4.802 | 12.5 | 43.6 | null | 7+8 | 4+12 |
| JimaiC70218 | 0 | 1.43 | 55.407 | 18.707 | 5.421 | 11.65 | 38.4 | null | 7+8 | 4+12 |
| JimaiC70223 | 1 | 1.34 | 57.579 | 22.165 | 5.959 | 11.79 | 38.3 | null | 7+8 | 4+12 |
| JimaiC70228 | 1 | 1.89 | 63.817 | 16.4 | 4.6 | 11.1 | 35 | null | 7+8 | 4+12 |
| JimaiC70231 | 1 | 1.53 | 60.69 | 17.164 | 6.07 | 11.58 | 40.1 | null | 7+8 | 4+12 |
| JimaiC70241 | 1 | 1.53 | 53.987 | 16.541 | 5.936 | 12.47 | 43.4 | 1 | 7+8 | 4+12 |
| JimaiC70245 | 1 | 1.29 | 50.332 | 16.523 | 5.427 | 11.28 | 38.2 | null | 7+8 | 4+12 |
| JimaiC70247 | 1 | 1.33 | 48.855 | 15.1 | 5.2 | 14.25 | 51.9 | 1 | 7+8 | 4+12 |
| JimaiC70285 | 1 | 2.02 | 69.06 | 22.456 | 11.421 | 11.47 | 37.7 | null | 7+8 | 2,4+12 |
| JimaiC70298 | 0 | 2.36 | 86.931 | 12.49 | 4.868 | 10.85 | 33.5 | null | 7+8 | 4+12 |
| JimaiC70321 | 0 | 1.89 | 78.922 | 20.344 | 10.584 | 9.43 | 27.8 | 1 | 7+8 | 4+12 |
| JimaiC70356 | 0 | 2.17 | 86.271 | 19.097 | 9.864 | 11.44 | 37.1 | 1 | 7+8 | 4+12 |
| JimaiC70361 | 0 | 1.81 | 70.691 | 19.751 | 6.027 | 14.02 | 51.1 | 1 | 7+8 | 4+12 |
| JimaiC70365 | 0 | 1.41 | 64.073 | 21.894 | 6.201 | 11.1 | 34.9 | 1 | 7+8 | 4+12 |
| JimaiC70373 | 1 | 2.97 | 102.279 | 11.555 | 7.204 | 11.29 | 36.8 | 1 | 7+8 | 4+12 |
| JimaiC70421 | 1 | 2.62 | 102.053 | 18.395 | 7.501 | 11.85 | 39.7 | 1 | 14+15 | 4+12 |
| JimaiC70445 | 1 | 2.86 | 110.716 | 18.56 | 7.323 | 10.84 | 32.3 | 1 | 14+15 | 4+12 |
| JimaiC70459 | 1 | 1.21 | 47.936 | 22.676 | 3.612 | 10.6 | 33.2 | 1 | 7+8 | 4+12 |
| JimaiC70483 | 1 | 1.27 | 47.785 | 17.96 | 3.663 | 11.87 | 39.4 | 1 | 7+8 | 4+12 |
| JimaiC70509 | 1 | 3.03 | 115.491 | 16.989 | 10.829 | 11.13 | 36.8 | 1 | 7+8 | 2+12 |
| JimaiD101 | 1 | 2.74 | 108.104 | 15.989 | 5.96 | 11.81 | 40.3 | 1 | 7+8 | 4+12 |
| JimaiD102 | 0 | 5.29 | 202.277 | 14.319 | 9.703 | 11.61 | 41.8 | 1 | 7+8 | 2+12 |
| JimaiD103 | 0 | 2.64 | 105.913 | 15.515 | 4.644 | 11.51 | 42.3 | 1 | 7+8 | 5+10 |
| JimaiD104 | 0 | 3.01 | 115.57 | 15.272 | 5.336 | 10.16 | 33.9 | 1 | 7+8 | 2+12 |
| JimaiD105 | 0 | 2.99 | 109.856 | 15.935 | 5.894 | 10.45 | 35.7 | 1 | 7+8 | 2+12 |
| JimaiD106 | 1 | 3.11 | 116.808 | 14.477 | 5.325 | 8.85 | 34.8 | 1 | 7+8 | 2+12 |
| JimaiD107 | 1 | 2.31 | 101.776 | 18.357 | 5.826 | 10.66 | 36.1 | 1 | 7+8 | 2+12 |
| JimaiD108 | 1 | 1.68 | 61.818 | 26.132 | 6.782 | 8.34 | 33.2 | 1 | 14+15 | 2+12 |
| JimaiD109 | 1 | 5.48 | 201.092 | 14.299 | 11.484 | 10.19 | 34.4 | 1 | 14+15 | 2+12 |
| JimaiD111 | 1 | 2.69 | 87.364 | 10.083 | 3.542 | 11.89 | 41.1 | 1 | 14+15 | 5+10 |
| JimaiD113 | 1 | 2.58 | 91.505 | 19.098 | 4.932 | 12.44 | 42.8 | 1 | 7+9 | 5+10 |
| JimaiD116 | 0 | 2.79 | 104.459 | 16.41 | 3.754 | 12.97 | 45.1 | 1 | 7+9 | 2+12 |
| JimaiD117 | 0 | 3.6 | 171.802 | 30.553 | 8.403 | 12.86 | 43.7 | 1 | 7+8 | 2+12 |
| JimaiD118 | 0 | 2.7 | 129.869 | 22.331 | 6.59 | 13.92 | 48 | 1 | 7+8 | 2+12 |
| JimaiD119 | 1 | 4.2 | 184.289 | 19.308 | 14.624 | 12.69 | 43.4 | 1 | 7+8 | 2+12 |
| JimaiD120 | 1 | 4.86 | 193.539 | 23.81 | 13.989 | 13.23 | 45.4 | 1 | 14+15 | 5+10 |
| JimaiD121 | 1 | 3.6 | 140.688 | 16.922 | 6.263 | 11.11 | 36.6 | 2* | 7+9 | 5+10 |
| JimaiD122 | 0 | 2.82 | 103.273 | 15.139 | 5.094 | 11.76 | 39.3 | 1 | 7+8 | 5+10 |
| JimaiD123 | 0 | 3.16 | 122.844 | 14.48 | 5.359 | 11.7 | 38.6 | 1 | 7+8 | 5+10 |
| JimaiD124 | 0 | 3.08 | 109.688 | 15.336 | 4.621 | 10.78 | 35.1 | 1 | 14+15 | 2+12 |
| JimaiH101 | 1 | 1.68 | 80.759 | 23.976 | 4.173 | 14.21 | 49.9 | 1 | 14+15 | 2+12 |
| JimaiH102 | 0 | 1.54 | 63.859 | 21.909 | 4.06 | 14.07 | 48.5 | 1 | 7+8 | 5+10 |
| JimaiH105 | 0 | 1.9 | 80.617 | 21.182 | 4.384 | 13.5 | 46.5 | 1 | 7+8 | 5+10 |
| JimaiH106 | 1 | 1.56 | 61.64 | 20.268 | 4.951 | 10.51 | 38.9 | 1 | 7+8 | 5+10 |
| JimaiH107 | 1 | 2.16 | 76.83 | 15.586 | 3.572 | 13.84 | 48.5 | 2* | 14+15 | 2+12 |
| JimaiH108 | 1 | 1.82 | 60.41 | 9.884 | 2.946 | 10.37 | 39.7 | 2* | 7+8 | 2+12 |
| JimaiH109 | 1 | 3.14 | 129.959 | 21.741 | 6 | 13.38 | 46.7 | 1 | 7+8 | 2+12 |
| JimaiH110 | 1 | 1.9 | 74.445 | 15.632 | 4.372 | 10.45 | 39.6 | 1 | 7+8 | 2+12 |
| JimaiH111 | 0 | 1.79 | 65.821 | 17.204 | 2.885 | 12.04 | 42.5 | 1 | 7+8 | 2+12 |
| JimaiH112 | 0 | 1.63 | 65.216 | 18.141 | 3.782 | 11.07 | 40.4 | 1 | 14+15 | 2+12 |
| JimaiH113 | 0 | 2.1 | 84.129 | 18.581 | 2.973 | 12.4 | 42.6 | 1 | 14+15 | 5+10 |
| JimaiH114 | 0 | 1.45 | 55.453 | 16.281 | 3.257 | 9.92 | 38.4 | 1 | 7+8 | 5+10 |
| JimaiH117 | 0 | 3.63 | 135.704 | 20.188 | 4.946 | 12.18 | 40.9 | 1 | 7+8 | 5+10 |
| JimaiH118 | 0 | 2.28 | 86.238 | 21.191 | 3.918 | 13.05 | 45.4 | 1 | 7+8 | 2+12 |
| JimaiH120 | 1 | 3.49 | 130.656 | 23.348 | 10.574 | 11.84 | 39.4 | 1 | 7+8 | 2+12 |
| JimaiH122 | 0 | 1.93 | 74.204 | 18.096 | 4.81 | 11.69 | 38.5 | 1 | 7+8 | 2+12 |
| JimaiH123 | 0 | 2.08 | 82.496 | 19.779 | 4.745 | 11.65 | 38.2 | 1 | 7+8 | 2+12 |
| JimaiH124 | 0 | 2.07 | 78.41 | 16.058 | 4.802 | 11.65 | 38.2 | 1 | 14+15 | 5+10 |
| JimaiH125 | 0 | 3.42 | 125.2 | 17.214 | 5.019 | 12.06 | 39.2 | 1 | 7+8 | 2+12 |
| JimaiT102 | 0 | 1.68 | 62.574 | 18.385 | 3.308 | 14.56 | 50.1 | 1 | 7+8 | 5+10 |
| JimaiT103 | 0 | 1.82 | 64.978 | 17.802 | 3.996 | 14.79 | 51.5 | 1 | 14+15 | 2+12 |
| JimaiT104 | 0 | 1.43 | 54.558 | 18.072 | 3.163 | 13.9 | 47.9 | 1 | 14+15 | 5+10 |
| JimaiT105 | 1 | 1.9 | 75.151 | 17.816 | 3.897 | 13.77 | 47.4 | 1 | 7+9 | 5+10 |
| JimaiT108 | 1 | 1.57 | 60.065 | 14.837 | 2.997 | 11.18 | 41.8 | 1 | 7+9 | 5+10 |
| JimaiT109 | 1 | 2.77 | 114.2 | 20.864 | 7.007 | 14.31 | 49.3 | 1 | 7+8 | 5+10 |
| JimaiT110 | 1 | 2.03 | 75.28 | 16.758 | 4.11 | 10.7 | 40.6 | 1 | 7+8 | 5+10 |
| JimaiT111 | 1 | 2.4 | 75.703 | 12.375 | 0.337 | 12.76 | 43.5 | 2* | 14+15 | 2+12 |
| JimaiT112 | 0 | 2 | 71.425 | 14.451 | 3.566 | 11.81 | 40.5 | 1 | 7+9 | 2+12 |
| JimaiT118 | 0 | 2.61 | 92.226 | 16.957 | 4.564 | 13.3 | 43.5 | 2* | 7+9 | 2+12 |
| JimaiT120 | 1 | 4.23 | 161.178 | 19.316 | 11.057 | 13.07 | 45.1 | 1 | 7+8 | 2+12 |
| JimaiT216 | 0 | 1.78 | 64.673 | 14.597 | 3.17 | 10.94 | 42 | 1 | 7+8 | 5+10 |
| JimaiT30005 | 0 | 2.59 | 97.032 | 18.493 | 9.782 | 12.82 | 43.6 | 1 | 7+8 | 5+10 |
| JimaiT40097 | 1 | 2.06 | 79.038 | 18.092 | 6.213 | 10.6 | 35.7 | 1 | 7+8 | 2+12 |
| JimaiT40098 | 1 | 2.29 | 75.248 | 13.42 | 5.432 | 11.03 | 38.2 | 1 | 14+15 | 2+12 |
| JimaiT40103 | 1 | 1.97 | 71.5 | 17.083 | 4.687 | 10.92 | 37.3 | 1 | 14+15 | 2+12 |
| JimaiT40271 | 1 | 3.52 | 142.178 | 15.333 | 12.24 | 10.34 | 34 | null | 7+9 | 2+12 |
| JimaiT40284 | 0 | 2.31 | 86.254 | 15.267 | 8.001 | 12.81 | 45.2 | 1 | 7+9 | 2+12 |
| JimaiT40362 | 1 | 3.45 | 146.615 | 19.069 | 11.643 | 10.38 | 34.1 | 1 | 14+15 | 2+12 |
| JimaiT40368 | 1 | 4.23 | 167.428 | 15.699 | 11.533 | 11.18 | 37.6 | 1 | 14+15 | 2+12 |

**Sig N-ter Rep-region**

1 MKVFILALLALTATTAIAQLESTCSQGFGQCQHQQ.PGQQQLLEQMKPCV 49

2 MKVFILALLALTATTAIAQLESTCSQGFGQCQHQQ.PGQQQLLEQMKPCV 49

3 MKVFILALLALTATTAIAQLESTCSQGFGQCQHQQ.PGQQQLLEQMKPCV 49

4 MKVFILALLALTATTAIAQLESTCSQGFGQCQHQQ.PGQQQLLEQMKPCV 49

5 MKVFILALLALTATTAIAQLESTCSQGFGQCQHQQ.PGQQQLLEQMKPCV 49

6 MKVFILALLALTATTAIAQLESTCSQGFGQCQHQQ.PGQQQLLEQMKPCV 49

7 MKVFILALLALTATTAIAQLESTCSQGFGQCQHQQ.PGQQQLLEQMKPCV 49

8 MKVFILALLALTATTAIAQLESTCSQGFGQCQHQQ.PGQQQLLEQMKPCV 49

9 MKVFILALLALTATTAIAQLESTCSQGFGQCQHQQ.PGQQQLLEQMKPCV 49

10 MKVFILALLALTATTAIAQLESTCSQGFGQCQHQQ.PGQQQLLEQMKPCV 49

11 MKVFILALLALTATTAIAQLESTCSQGFGQCQQQQ.PGQQQLLEQMKPCV 49

12 MKVFILALLALTATTAIAQLESTCSQGFGQCQQQQ.PGQQQLLEQMKPCV 49

13 MKVFILALLALTATTAIAQLESTCSQGFGQCQQQQ.PGQQQLLEQMKPCV 49

14 MKVFILALLALTATTAIAQLESTCSQGFGQCQQQQ.PGQQQLLEQMKPCV 49

15 MKVFILALLALTATTAIAQLESTCSQGFGQCQQQQ.PGQQQLLEQMKPCV 49

16 MKVFILALLALTATTAIAQLESTCSQGFGQCQQQQQPGQQQLLEQMKPCV 50

17 MKVFILALLALTATTAIAQLESTCSQGFGQCQQQQQPGQQQLLEQMKPCV 50

18 MKVFILALLALTATTAIAQLESTCSQGFGQCQQQQQPGQQQLLEQMKPCV 50

19 MKVFILALLALTATTAIAQLESTCSQGFGQCQQQQQPGQQQLLEQMKPCV 50

1 AFLQQQCSPVRMPFLQIQVQQLSSCQIMQYQCCQQLAQIPERIRCHAIHS 99

2 AFLQQQCSPVRMPFLQIQVQQLSSCQIMQYQCCQQLAQIPERIRCHAIHS 99

3 AFLQQQCSPVRMPFLQIQVQQLSSCQIMQYQCCQQLAQIPERIRCHAIHS 99

4 AFLQQQCSPVRMPFLQIQVQQLSSCQIMQYQCCQQLAQIPERIRCHAIHS 99

5 AFLQQQCSPVRMPFLQIQVQQLSSCQIMQYQCCQQLAQIPERIRCHAIHS 99

6 AFLQQQCSPVRMPFLQIQVQQLSSCQIMQYQCCQQLAQIPERIRCHAIHS 99

7 AFLQQQCSPVRMPFLQIQVQQLSSCQIMQYQCCQQLAQIPERIRCHAIHS 99

8 AFLQQQCSPVRMPFLQIQVQQLSSCQIMQYQCCQQLAQIPERIRCHAIHS 99

9 AFLQQQCSPVRMPFLQIQVQQLSSCQIMQYQCCQQLAQIPERIRCHAIHS 99

10 AFLQQQCSPVRMPFLQIQVQQLSSCQIMQYQCCQQLAQIPERIRCHAIHS 99

11 AFLQQQCSPVRMPFLQIQVQQLSSCHIMQYQCCQQLAQIPERIRCHAIHS 99

12 AFLQQQCSPVRMPFLQIQVQQLSSCHIMQYQCCQQLAQIPERIRCHAIHS 99

13 AFLQQQCSPVRMPFLQIQVQQLSSCHIMQYQCCQQLAQIPERIRCHAIHS 99

14 AFLQQQCSPVRMPFLQIQVQQLSSCHIMQYQCCQQLAQIPERIRCHAIHS 99

15 AFLQQQCSPVRMPFLQIQVQQLSSCHIMQYQCCQQLAQIPERIRCHAIHS 99

16 AFLQQQCSPVRMPFLQIQVQQLSSCHIMQYQCCQQLAQIPERIRCHAIHS 100

17 AFLQQQCSPVRMPFLQIQVQQLSSCHIMQYQCCQQLAQIPERIRCHAIHS 100

18 AFLQQQCSPVRMPFLQIQVQQLSSCHIMQYQCCQQLAQIPERIRCHAIHS 100

19 AFLQQQCSPVRMPFLQIQVQQLSSCHIMQYQCCQQLAQIPERIRCHAIHS 100

1 VVEAIMQQQPQQQWQEPQQQAQHKSIRMLLENLSLMCSISVPVQCQQQQQ 149

2 VVEAIMQQQPQQQWQEPQQQAQHKSIRMLLENLSLMCSISVPVQCQQQQQ 149

3 VVEAIMQQQPQQQWQEPQQQAQHKSIRMLLENLSLMCSISVPVQCQQQQQ 149

4 VVEAIMQQQPQQQWQEPQQQAQHKSIRMLLENLSLMCSISVPVQCQQQQQ 149

5 VVEAIMQQQPQQQWQEPQQQAQHKSIRMLLENLSLMCSISVPVQCQQQQQ 149

6 VVEAIMQQQPQQQWQEPQQQAQHKSIRMLLENLSLMCSISVPVQCQQQQQ 149

7 VVEAIMQQQPQQQWQEPQQQAQHKSIRMLLENLSLMCSISVPVQCQQQQQ 149

8 VVEAIMQQQPQQQWQEPQQQAQHKSIRMLLENLSLMCSISVPVQCQQQQQ 149

9 VVEAIMQQQPQQQWQEPQQQAQHKSIRMLLENLSLMCSISVPVQCQQQQQ 149

10 VVEAIMQQQPQQQWQEPQQQAQHKSIRMLLENLSLMCSISVPVQCQQQQQ 149

11 VVEAIMQQQPQQQWQEPQQQAQHKSIRMLLENLSLMCSISVPVQCQQQQQ 149

12 VVEAIMQQQPQQQWQEPQQQAQHKSIRMLLENLSLMCSISVPVQCQQQQQ 149

13 VVEAIMQQQPQQQWQEPQQQAQHKSIRMLLENLSLMCSISVPVQCQQQQQ 149

14 VVEAIMQQQPQQQWQEPQQQAQHKSIRMLLENLSLMCSISVPVQCQQQQQ 149

15 VVEAIMQQQPQQQWQEPQQQAQHKSIRMLLENLSLMCSISVPVQCQQQQQ 149

16 VVEAIMQQQPQQQWQEPQQQAQHKSIRMLLENLSLMCSISVPVQCQQQQQ 150

17 VVEAIMQQQPQQQWQEPQQQAQHKSIRMLLENLSLMCSISVPVQCQQQQQ 150

18 VVEAIMQQQPQQQWQEPQQQAQHKSIRMLLENLSLMCSISVPVQCQQQQQ 150

19 VVEAIMQQQPQQQWQEPQQQAQHKSIRMLLENLSLMCSISVPVQCQQQQQ 150

1 LGQQQQQ*LQEQLTPCATFLQQQCSPMTVPFPQTLVDQPTSCQNVQHQCC 199

2 LGQQQQQ*LQEQLTPCATFLQQQCSPMTVPFPQTLVDQPTSCQNVQHQCC 199

3 LGQQQQQ*LQEQLTPCATFLQQQCSPMTVPFPQTLVDQPTSCQNVQHQCC 199

4 LGQQQQQ*LQEQLTPCATFLQQQCSPMTVPFPQTLVDQPTSCQNVQHQCC 199

5 LGQQQQQ*LQEQLTPCATFLQQQCSPMTVPFPQTLVDQPTSCQNVQHQCC 199

6 LGQQQQQ*LQEQLTPCATFLQQQCSPMTVPFPQTLVDQPTSCQNVQHQCC 199

7 LGQQQQQ*LQEQLTPCATFLQQQCSPMTVPFPQTLVDQPTSCQNVQHQCC 199

8 LGQQQQQ*LQEQLTPCATFLQQQCSPMTVPFPQTLVDQPTSCQNVQHQCC 199

9 LGQQQQQ*LQEQLTPCATFLQQQCSPMTVPFPQTLVDQPTSCQNVQHQCC 199

10 LGQQQQQ*LQEQLTPCATFLQQQCSPMTVPFPQTLVDQPTSCQNVQHQCC 199

11 LGQQQKQQLQEQLTPCATFLQQQCSPMTVPFPQTPVDQPTSCQNVQHQCC 199

12 LGQQQKQQLQEQLTPCATFLQQQCSPMTVPFPQTPVDQPTSCQNVQHQCC 199

13 LGQQQKQQLQEQLTPCATFLQQQCSPMTVPFPQTPVDQPTSCQNVQHQCC 199

14 LGQQQKQQLQEQLTPCATFLQQQCSPMTVPFPQTPVDQPTSCQNVQHQCC 199

15 LGQQQKQQLQEQLTPCATFLQQQCSPMTVPFPQTPVDQPTSCQNVQHQCC 199

16 LGQQQKQQLQEQLTPCATFLQQQCSPMTVPFPQTPVDQPTSCQNVQHQCC 200

17 LGQQQKQQLQEQLTPCATFLQQQCSPMTVPFPQTPVDQPTSCQNVQHQCC 200

18 LGQQQKQQLQEQLTPCATFLQQQCSPMTVPFPQTPVDQPTSCQNVQHQCC 200

19 LGQQQKQQLQEQLTPCATFLQQQCSPMTVPFPQTPVDQPTSCQNVQHQCC 200

**C-ter**

1 RQLSHIPEQFRCQAIHNVAEAIRQQRPQQQWQGMYQAQQPAQLESIRMSL 249

2 RQLSHIPEQFRCQAIHNVAEAIRQQRPQQQWQGMYQAQQPAQLESIRMSL 249

3 RQLSHIPEQFRCQAIHNVAEAIRQQRPQQQWQGMYQAQQPAQLESIRMSL 249

4 RQLSHIPEQFRCQAIHNVAEAIRQQRPQQQWQGMYQAQQPAQLESIRMSL 249

5 RQLSHIPEQFRCQAIHNVAEAIRQQRPQQQWQGMYQAQQPAQLESIRMSL 249

6 RQLSHIPEQFRCQAIHNVAEAIRQQRPQQQWQGMYQAQQPAQLESIRMSL 249

7 RQLSHIPEQFRCQAIHNVAEAIRQQRPQQQWQGMYQAQQPAQLESIRMSL 249

8 RQLSHIPEQFRCQAIHNVAEAIRQQRPQQQWQGMYQAQQPAQLESIRMSL 249

9 RQLSHIPEQFRCQAIHNVAEAIRQQRPQQQWQGMYQAQQPAQLESIRMSL 249

10 RQLSHIPEQFRCQAIHNVAEAIRQQRPQQQWQGMYQAQQPAQLESIRMSL 249

11 RQLSHIPEQFRCQAIHNVAEAIRQQRPQQQWQGMYQAQQPAQLESIRMSL 249

12 RQLSHIPEQFRCQAIHNVAEAIRQQRPQQQWQGMYQAQQPAQLESIRMSL 249

13 RQLSHIPEQFRCQAIHNVAEAIRQQRPQQQWQGMYQAQQPAQLESIRMSL 249

14 RQLSHIPEQFRCQAIHNVAEAIRQQRPQQQWQGMYQAQQPAQLESIRMSL 249

15 RQLSHIPEQFRCQAIHNVAEAIRQQRPQQQWQGMYQAQQPAQLESIRMSL 249

16 RQLSHIPEQFRCQAIHNVAEAIRQQRPQQQWQGMYQAQQPAQLESIRMSL 250

17 RQLSHIPEQFRCQAIHNVAEAIRQQRPQQQWQGMYQAQQPAQLESIRMSL 250

18 RQLSHIPEQFRCQAIHNVAEAIRQQRPQQQWQGMYQAQQPAQLESIRMSL 250

19 RQLSHIPEQFRCQAIHNVAEAIRQQRPQQQWQGMYQAQQPAQLESIRMSL 250

1 QALRSMCSIYIPVQCPAPTAYNIPMVATYPGGAY 283

2 QALRSMCSIYIPVQCPAPTAYNIPMVATYPGGAY 283

3 QALRSMCSIYIPVQCPAPTAYNIPMVATYPGGAY 283

4 QALRSMCSIYIPVQCPAPTAYNIPMVATYPGGAY 283

5 QALRSMCSIYIPVQCPAPTAYNIPMVATYPGGAY 283

6 QALRSMCSIYIPVQCPAPTAYNIPMVATYPGGAY 283

7 QALRSMCSIYIPVQCPAPTAYNIPMVATYPGGAY 283

8 QALRSMCSIYIPVQCPAPTAYNIPMVATYPGGAY 283

9 QALRSMCSIYIPVQCPAPTAYNIPMVATYPGGAY 283

10 QALRSMCSIYIPVQCPAPTAYNIPMVATYPGGAY 283

11 QALRSMCSIYIPVQCPAPTAYNIPMVATYPGGAY 283

12 QALRSMCSIYIPVQCPAPTAYNIPMVATYPGGAY 283

13 QALRSMCSIYIPVQCPAPTAYNIPMVATYPGGAY 283

14 QALRSMCSIYIPVQCPAPTAYNIPMVATYPGGAY 283

15 QALRSMCSIYIPVQCPAPTAYNIPMVATYPGGAY 283

16 QALRSMCSIYIPVQCPAPTAYNIPMVATYPGGAY 284

17 QALRSMCSIYIPVQCPAPTAYNIPMVATYPGGAY 284

18 QALRSMCSIYIPVQCPAPTAYNIPMVATYPGGAY 284

19 QALRSMCSIYIPVQCPAPTAYNIPMVATYPGGAY 284

**Supplementary Figure 1. Alignment of the translated amino acid sequences of type-b ALP genes on chromosome 7AS.**

▲: Position of the cysteine residues; *: stop codon; Sig: Signal peptide; N-ter: N-terminus; C-ter: C-terminus; Rep-region: Repetitive region. 1, Chinese Spring; 2, Eagle Rock; 3, Jimai13J394; 4, Jimai13J409; 5, Jimai13J492; 6, Jimai13P406; 7, Jimai0860229; 8, Living Stone; 9, Westonia; 10, Wyalkatchem; 11, Jimai13J494; 12, Jimai13P414; 13, Jimai23; 14, Jimai24; 15, Jimai44; 16, Kauz; 17, Yitpi; 18, Gregory; 19, Chara.

Sig N-ter Rep-region

1 MKVFILALLALTATTAIAQLETTCSQGFGQYQQQQQPGQRQLLEQMKPCV 50

2 MKVFILALLALTATTAIAQLETTCSQGFGQYQQQQQPGQRQLLEQMKPCV 50

3 MKVFILALLALTATTAIAQLETTCSQGFGQYQQQQQPGQRQLLEQMKPCV 50

4 MKVFILALLALTATTAIAQLETTCSQGFGQYQQQQQPGQRQLLEQMKPCV 50

5 MKVFILALLALTATTAIAQLETTCSQGFGQYQQQQQPGQRQLLEQMKPCV 50

6 MKVFILALLALTATTAIAQLETTCSQGFGQYQQQQQPGQRQLLEQMKPCV 50

7 MKVFILALLALTATTAIAQLETTCSQGFGQYQQQQQPGQRQLLEQMKPCV 50

8 MKVFILALLALTATTAIAQLETTCSQGFGQYQQQQQPGQRQLLEQMKPCV 50

9 MKVFILALLALTATTAIAQLETTCSQGFGQYQQQQQPGQRQLLEQMKPCV 50

10 MKVFILALLALTATTAIAQLETTCSQGFGQYQQQQQPGQRQLLEQMKPCV 50

11 MKVFILALLALTATTAIAQLETTCSQGFGQYQQQQQPGQRQLLEQMKPCV 50

12 MKVFILALLALTATTAIAQLETTCSQGFGQYQQQQQPGQRQLLEQMKPCV 50

13 MKVFILALLALTATTAIAQLETTCSQGFGQYQQQQQPGQRQLLEQMKPCV 50

14 MKVFILALLALTATTAIAQLETTCSQGFGQYQQQQQPGQRQLLEQMKPCV 50

15 MKVFILALLALTATTAIAQLETTCSQGFGQYQQQQQPGQRQLLEQMKPCV 50

16 MKVFILALLALTATTAIAQLETTCSQGFGQYQQQQQPGQRQLLEQMKPCV 50

17 MKVFILALLALTATTAIAQLETTCSQGFGQYQQQQQPGQRQLLEQMRPCV 50

18 MKVFILALLALTATTAIAQLETTCSQGFGQYQQQQQPGQRQLLEQMRPCV 50

19 MKVFILALLALTATTAIAQLETTCSQGFGQYQQQQQPGQRQLLEQMRPCV 50

1 AFLQQQCRPLRMPFLQTQVEQLSSCQIVQHQCCQQLAQIPERIRCHAIHS 100

2 AFLQQQCRPLRMPFLQTQVEQLSSCQIVQHQCCQQLAQIPERIRCHAIHS 100

3 AFLQQQCRPLRMPFLQTQVEQLSSCQIVQHQCCQQLAQIPERIRCHAIHS 100

4 AFLQQQCRPLRMPFLQTQVEQLSSCQIVQHQCCQQLAQIPERIRCHAIHS 100

5 AFLQQQCRPLRMPFLQTQVEQLSSCQIVQHQCCQQLAQIPERIRCHAIHS 100

6 AFLQQQCRPLRMPFLQTQVEQLSSCQIVQHQCCQQLAQIPERIRCHAIHS 100

7 AFLQQQCRPLRMPFLQTQVEQLSSCQIVQHQCCQQLAQIPERIRCHAIHS 100

8 AFLQQQCRPLRMPFLQTQVEQLSSCQIVQHQCCQQLAQIPERIRCHAIHS 100

9 AFLQQQCRPLRMPFLQTQVEQLSSCQIVQHQCCQQLAQIPERIRCHAIHS 100

10 AFLQQQCRPLRMPFLQTQVEQLSSCQIVQHQCCQQLAQIPERIRCHAIHS 100

11 AFLQQQCRPLRMPFLQTQVEQLSSCQIVQHQCCQQLAQIPERIRCHAIHS 100

12 AFLQQQCRPLRMPFLQTQVEQLSSCQIVQHQCCQQLAQIPERIRCHAIHS 100

13 AFLQQQCRPLRMPFLQTQVEQLSSCQIVQHQCCQQLAQIPERIRCHAIHS 100

14 AFLQQQCRPLRMPFLQTQVEQLSSCQIVQHQCCQQLAQIPERIRCHAIHS 100

15 AFLQQQCRPLRMPFLQTQVEQLSSCQIVQHQCCQQLAQIPERIRCHAIHS 100

16 AFLQQQCRPLRMPFLQTQVEQLSSCQIVQHQCCQQLAQIPERIRCHAIHS 100

17 AFLQQQCRPLRMPFLQTQVEQLSSCQIVQYQCCQQLAQIPEQIRCHAIHN 100

18 AFLQQQCRPLRMPFLQTQVEQLSSCQIVQYQCCQQLAQIPEQIRCHAIHN 100

19 AFLQQQCRPLRMPFLQTQVEQLSSCQIVQYQCCQQLAQIPEQIRCHAIHN 100

1 VVEAIMQQQSQQQWQERQQQAQHKSMRMLLENLSLMCNIYVPVQCQQQQQ 150

2 VVEAIMQQQSQQQWQERQQQAQHKSMRMLLENLSLMCNIYVPVQCQQQQQ 150

3 VVEAIMQQQSQQQWQERQQQAQHKSMRMLLENLSLMCNIYVPVQCQQQQQ 150

4 VVEAIMQQQSQQQWQERQQQAQHKSMRMLLENLSLMCNIYVPVQCQQQQQ 150

5 VVEAIMQQQSQQQWQERQQQAQHKSMRMLLENLSLMCNIYVPVQCQQQQQ 150

6 VVEAIMQQQSQQQWQERQQQAQHKSMRMLLENLSLMCNIYVPVQCQQQQQ 150

7 VVEAIMQQQSQQQWQERQQQAQHKSMRMLLENLSLMCNIYVPVQCQQQQQ 150

8 VVEAIMQQQSQQQWQERQQQAQHKSMRMLLENLSLMCNIYVPVQCQQQQQ 150

9 VVEAIMQQQSQQQWQERQQQAQHKSMRMLLENLSLMCNIYVPVQCQQQQQ 150

10 VVEAIMQQQSQQQWQERQQQAQHKSMRMLLENLSLMCNIYVPVQCQQQQQ 150

11 VVEAIMQQQSQQQWQERQQQAQHKSMRMLLENLSLMCNIYVPVQCQQQQQ 150

12 VVEAIMQQQSQQQWQERQQQAQHKSMRMLLENLSLMCNIYVPVQCQQQQQ 150

13 VVEAIMQQQSQQQWQERQQQAQHKSMRMLLENLSLMCNIYVPVQCQQQQQ 150

14 VVEAIMQQQSQQQWQERQQQAQHKSMRMLLENLSLMCNIYVPVQCQQQQQ 150

15 VVEAIMQQQSQQQWQERQQQAQHKSMRMLLENLSLMCNIYVPVQCQQQQQ 150

16 VVEAIMQQQSQQQWQERQQQAQHKSMRMLLENLSLMCNIYVPVQCQQQQQ 150

17 VVEAIMQQQSQQQRQERQQQAQHKSMRMLLENLSLMCNIYVPIQCQQQQQ 150

18 VVEAIMQQQSQQQRQERQQQAQHKSMRMLLENLSLMCNIYVPIQCQQQQQ 150

19 VVEAIMQQQSQQQRQERQQQAQHKSMRMLLENLSLMCNIYVPIQCQQQQQ 150

1 MGQQQQQQQLQEQLTPCATFLQHQCSPVTVPFPQIPVDQPTSCQNVQHQC 200

2 MGQQQQQQQLQEQLTPCATFLQHQCSPVTVPFPQIPVDQPTSCQNVQHQC 200

3 MGQQQQQQQLQEQLTPCATFLQHQCSPVTVPFPQIPVDQPTSCQNVQHQC 200

4 MGQQQQQQQLQEQLTPCATFLQHQCSPVTVPFPQIPVDQPTSCQNVQHQC 200

5 MGQQQQQQQLQEQLTPCATFLQHQCSPVTVPFPQIPVDQPTSCQNVQHQC 200

6 MGQQQQQQQLQEQLTPCATFLQHQCSPVTVPFPQIPVDQPTSCQNVQHQC 200

7 MGQQQQQQQLQEQLTPCATFLQHQCSPVTVPFPQIPVDQPTSCQNVQHQC 200

8 MGQQQQQQQLQEQLTPCATFLQHQCSPVTVPFPQIPVDQPTSCQNVQHQC 200

9 MGQQQQQQQLQEQLTPCATFLQHQCSPVTVPFPQIPVDQPTSCQNVQHQC 200

10 MGQQQQQQQLQEQLTPCATFLQHQCSPVTVPFPQIPVDQPTSCQNVQHQC 200

11 MGQQQQQQQLQEQLTPCATFLQHQCSPVTVPFPQIPVDQPTSCQNVQHQC 200

12 MGQQQQQQQLQEQLTPCATFLQHQCSPVTVPFPQIPVDQPTSCQNVQHQC 200

13 MGQQQQQQQLQEQLTPCATFLQHQCSPVTVPFPQIPVDQPTSCQNVQHQC 200

14 MGQQQQQQQLQEQLTPCATFLQHQCSPVTVPFPQIPVDQPTSCQNVQHQC 200

15 MGQQQQQQQLQEQLTPCATFLQHQCSPVTVPFPQIPVDQPTSCQNVQHQC 200

16 MGQQQQQQ.LQEQLTPCATFLQHQCSPVTVPFPQIPVDQPTSCQNVQHQC 199

17 LGQQQQQQ.LQEQLTPCATFLQHQCSPVTVPFPQIPVDQPTSCQNVQHQC 199

18 LGQQQQQQ.LQEQLTPCATFLQHQCSPVTVPFPQIPVDQPTSCQNVQHQC 199

19 LGQQQQQQ.LQEQLTPCATFLQHQCSPVTVPFPQIPVDQPTSCQNVQHQC 199

**C-ter**

1 CRQLSQIPEQFRCQAIHNVAEAIRQQQPQQQWQGMYQPQQPAQHESIRMS 250

2 CRQLSQIPEQFRCQAIHNVAEAIRQQQPQQQWQGMYQPQQPAQHESIRMS 250

3 CRQLSQIPEQFRCQAIHNVAEAIRQQQPQQQWQGMYQPQQPAQHESIRMS 250

4 CRQLSQIPEQFRCQAIHNVAEAIRQQQPQQQWQGMYQPQQPAQHESIRMS 250

5 CRQLSQIPEQFRCQAIHNVAEAIRQQQPQQQWQGMYQPQQPAQHESIRMS 250

6 CRQLSQIPEQFRCQAIHNVAEAIRQQQPQQQWQGMYQPQQPAQHESIRMS 250

7 CRQLSQIPEQFRCQAIHNVAEAIRQQQPQQQWQGMYQPQQPAQHESIRMS 250

8 CRQLSQIPEQFRCQAIHNVAEAIRQQQPQQQWQGMYQPQQPAQHESIRMS 250

9 CRQLSQIPEQFRCQAIHNVAEAIRQQQPQQQWQGMYQPQQPAQHESIRMS 250

10 CRQLSQIPEQFRCQAIHNVAEAIRQQQPQQQWQGMYQPQQPAQHESIRMS 250

11 CRQLSQIPEQFRCQAIHNVAEAIRQQQPQQQWQGMYQPQQPAQHESIRMS 250

12 CRQLSQIPEQFRCQAIHNVAEAIRQQQPQQQWQGMYQPQQPAQHESIRMS 250

13 CRQLSQIPEQFRCQAIHNVAEAIRQQQPQQQWQGMYQPQQPAQHESIRMS 250

14 CRQLSQIPEQFRCQAIHNVAEAIRQQQPQQQWQGMYQPQQPAQHESIRMS 250

15 CRQLSQIPEQFRCQAIHNVAEAIRQQQPQQQWQGMYQPQQPAQHESIRMS 250

16 CRQLSQIPEQFRCQAIHNVAEAIRQQQPQQQWQGMYQPQQPAQHESIRMS 249

17 CRQLSQIPEQFRCQAIHNVAEAIRQQQPQQQWQGMYQPQQPAQHESIRMS 249

18 CRQLSQIPEQFRCQAIHNVAEAIRQQQPQQQWQGMYQPQQPAQHESIRMS 249

19 CRQLSQIPEQFRCQAIHNVAEAIRQQQPQQQWQGMYQPQQPAQHESIRMS 249

1 LQALRSMCNIYIPVQCPAPTAYNIPMVATCTSGAC 285

2 LQALRSMCNIYIPVQCPAPTAYNIPMVATCTSGAC 285

3 LQALRSMCNIYIPVQCPAPTAYNIPMVATCTSGAC 285

4 LQALRSMCNIYIPVQCPAPTAYNIPMVATCTSGAC 285

5 LQALRSMCNIYIPVQCPAPTAYNIPMVATCTSGAC 285

6 LQALRSMCNIYIPVQCPAPTAYNIPMVATCTSGAC 285

7 LQALRSMCNIYIPVQCPAPTAYNIPMVATCTSGAC 285

8 LQALRSMCNIYIPVQCPAPTAYNIPMVATCTSGAC 285

9 LQALRSMCNIYIPVQCPAPTAYNIPMVATCTSGAC 285

10 LQALRSMCNIYIPVQCPAPTAYNIPMVATCTSGAC 285

11 LQALRSMCNIYIPVQCPAPTAYNIPMVATCTSGAC 285

12 LQALRSMCNIYIPVQCPAPTAYNIPMVATCTSGAC 285

13 LQALRSMCNIYIPVQCPAPTAYNIPMVATCTSGAC 285

14 LQALRSMCNIYIPVQCPAPTAYNIPMVATCTSGAC 285

15 LQALRSMCNIYIPVQCPAPTAYNIPMVATCTSGAC 285

16 LQALRSMCNIYIPVQCPAPTAYNIPMVATCTSGAC 284

17 LQALRSMCSIYIPVQCPAPTAYNIPMVATCTSGAC 284

18 LQALRSMCSIYIPVQCPAPTAYNIPMVATCTSGAC 284

19 LQALRSMCSIYIPVQCPAPTAYNIPMVATCTSGAC 284

**Supplementary Figure 2. Alignment of the translated amino acid sequences of type-b ALP genes on chromosome 4AL.**

▲: Position of the cysteine residues; *: stop codon; Sig: Signal peptide; N-ter: N-terminus; C-ter: C-terminus; Rep-region: Repetitive region. 1, Jimai13J492; 2, Kauz; 3, Jimai13J409; 4, Eagle Rock; 5, Chara; 6, Jimai13P406; 7, Jimai23; 8, Jimai24; 9, Jimai13J494; 10, Jimai13P414; 11, Yitpi; 12, Wyalkatchem; 13, Westonia; 14, Chinese Spring; 15, Gregory; 16, Living Stone; 17, Jimai44; 18, Jimai0860229; 19, Jimai13J394.
